# Surface functionalization by covalent immobilization of an innovative carvacrol derivative to avoid fungal biofilm formation

**DOI:** 10.1186/s13568-014-0091-2

**Published:** 2015-02-05

**Authors:** Aïcha Gharbi, Thibaut Legigan, Vincent Humblot, Sébastien Papot, Jean-Marc Berjeaud

**Affiliations:** 1 Ecologie & Biologie des Interactions - UMR CNRS 7267, Microbiologie de l’eau, Université de Poitiers, 1 Rue Georges Bonnet, TSA 51106, 86073 Poitiers Cedex 9, France; 2 UMR-CNRS 7285, Groupe Systèmes Moléculaires Programmés, Université de Poitiers, Poitiers, France; 3 UPMC Université Paris 06, UMR CNRS 7197, Laboratoire de Réactivité de Surface, Sorbonne Universités, Paris, France

**Keywords:** Antibiofilm material, *Candida albicans*, Carvacrol derivatives, Surface functionalization

## Abstract

Carvacrol, an aromatic terpenic compound, known to be antimicrobial was grafted onto gold surfaces via two strategies based on newly-synthesized cross-linkers involving either an ester bond which can be cleaved by microbial esterases, or a covalent ether link. Surface functionalizations were characterized at each step by reflection absorption infrared spectroscopy (RAIRS). The two functionalized gold samples both led to a loss of culturability of the yeast *Candida albicans,* higher than 65%, indicating that the activity of the freshly-designed surfaces was probably due to still covalently immobilized carvacrol. On the contrary, when a phenyl group replaced the terpenic moiety, the yeast culturability increased by about 30%, highlighting the specific activity of carvacrol grafted on the surfaces. Confocal microscopy analyses showed that the mode of action of the functionalized surfaces with the ester or the ether of carvacrol was, in both cases, fungicidal and not anti-adhesive. Finally, this study shows that covalently immobilization of terpenic compounds can be used to design promising antimicrobial surfaces.

**Electronic supplementary material:**

The online version of this article (doi:10.1186/s13568-014-0091-2) contains supplementary material, which is available to authorized users.

## Introduction

In recent years, hospital-acquired infections have considerably increased, concomitantly to the enhanced use of temporarily or permanently inserted indwelling medical devices (IMDs) (Ramage et al. 2009), thus becoming a real problem of public health. Fungi as well as bacteria can cause IMD-related infections. Today, invasive fungal infections are mainly associated to the *Candida* (80%) and *Aspergillus* genera (Cowen 2008; Silva et al. 2010). *C. albicans* being by far the most frequently isolated fungal species (Miceli et al. 2011) in such a way that it became a model study organism for this kind of infection (Gow et al. 2002; McCullough et al. 1996).

It is now clearly recognized that more than 65% of all human infections without any distinction are linked to the growth of biofilms (Pemàn et al. 2008; Pierce et al. 2008; Ramage et al. 2009). Biofilm formation is a complex process that can be summed up in four steps (Seneviratne et al. 2008): (i) the early initial phase or adherence phase followed by (ii) the intermediate phase of the biofilm growth, then (iii) the maturation phase of the newly formed biofilm and finally (iv) a dispersion phase of microorganisms from the biofilm (Pemàn et al. 2008). These biofilms are a major problem as the required dose of biocide to eradicate them can be higher than the highest therapeutic levels that can be administered to the patient (Ramage et al. 2009). Resulting from the presence of biofilms, the diagnosis and treatment of systemic candidal infections is often difficult (Walker et al. 2010).

One promising solution to avoid such infections can be to prevent biofilm formation from the beginning, by inhibiting the adherence phase that initiates the biofilm development cycle (von Eiff et al. 2005). In practice, several strategies currently exist. (i) Hospital good practices can be adopted (Pearson 1996). (ii) The lock therapy that consists in filling the lumen of a catheter still in place in the patient, with a highly-concentrated antimicrobial solution, for six to twelve hours, can also be used (Andris et al. 1998). This therapeutic approach has still limitations, in particular in the case of fungal infections (Hall and Farr 2004). (iii) Antimicrobial materials can also be developed. Indeed, non- functionalized biomaterials used to manufacture IMDs and catheters can be colonized by pathogenic microorganisms such as *C. albicans* (Costa et al. 2011). Modifying their surface, in order to make them unfavourable to microbial colonization, is an interesting and promising option (Tiller et al. 2001).

Material surface modifications can be performed in different ways in order to limit the establishment of microorganisms on the surface, to slow down their growth and thus to delay the biofilm formation (Roe et al. 2008). The coating approach by impregnation is today, the most developed one to design antimicrobial materials and the simplest one among all the technologies allowing a molecule release (Zhang et al. 2011). These surfaces suffer from a problem of antimicrobial activity depletion associated to the active substance release (Costa et al. 2011; Goddard and Hotchkiss 2007; Timofeeva and Kleshcheva 2011). To counteract this latter problem, the surface can also be functionalized by covalent grafting of the active molecule. Important achievements have been made during the last decade to covalently immobilized antimicrobial on surfaces (Costa et al. 2011; Goddard and Hotchkiss 2007; Timofeeva and Kleshcheva 2011). The inhibition of biofilm formation on a functionalized biomaterial may occur either through an anti-adhesive process (namely the reduction of microorganism adherence) or through a bacteriostatic action (namely avoiding cell multiplication on the surface in the presence of the antimicrobial agent) (Tenke et al. 2004) or finally through a bactericidal activity (namely by killing cells once in contact with the surface) (Aslam 2008). Different grafting strategies and types of antimicrobial agents have been more or less successfully tested such as heparin (Dwyer 2008), quaternary ammoniums (Abraham et al. 2002), or even antimicrobial peptides (*i.e.* Magainin I: (Humblot et al. 2009); Gramicidin A: (Yala et al. 2011)). In this field, one emergent trend consists in developing functionalized responsive materials from which the antimicrobial substance release is triggered by the microorganisms themselves, when approaching the surface. Indeed, as enzyme-sensitive linkages are introduced in such self-regulating systems, the active compound is only released in presence of the microorganisms that secrete the specific enzymes that can cleave the sensitive linkage in the construction (*e.g.* lipases (Komnatnyy et al. 2014)). Thus, the release of the active molecules can be tuned in function of the concentration of microorganisms surrounding the surface. This approach is supposed to increase the half-life of the biomolecule by avoiding its metabolization (Goddard and Hotchkiss 2007) or to limit the side-effects related to the molecule release that can lead to its accumulation in some vital tissues (*e.g.* brain, liver, spleen) (Costa et al. 2011).

Antimicrobial, antiseptic and antifungal and even anti-*Candida* properties of essential oils are often ascribed to their main volatile and aromatic terpenic components such as eugenol, thymol or carvacrol (CAR) (Braga et al. 2008; Pavel et al. 2010; Williams et al. 2011). Antifungal and anti-Candida biofilm properties of CAR have already been highlighted in a study from the laboratory (Dalleau et al. 2008). Regardless of the intensive research currently pursued on terpenes, their precise mechanisms of action remain slightly characterized (Gutierrez et al. 2010; Xu et al. 2008). In general, CAR has a fungicidal activity (namely by killing cells once in contact with the surface) on *C. albicans* (Ahmad et al. 2011; Braga et al. 2008).

In this work, two proofs of concept of innovative materials, functionalized with an original construction containing CAR covalently immobilized, was developed on gold surfaces. Indeed, CAR was attached to a specific spacer anchored on a gold surface through either an ester bound which could be cleaved by microorganisms’ esterases or an ether one which cannot be split. Thus, in the “ester” case, the antibiofilm activity should result from the release of CAR triggered by microorganisms approaching the surface making the surface repellent to them, whereas in the “ether” case, this activity should be associated to CAR, still attached to the surface. The antibiofilm activity of the new surfaces was studied by CFU counting as well as microscopic observations to understand the mode of action of such functionalized surfaces. The relevance of these two systems was tested on *C. albicans* in the present work.

## Materials and methods

### Microorganisms and growth conditions

The used strain *C. albicans* ATCC 3153 was grown for 48 h at 37°C on Sabouraud Glucose agar containing Chloramphenicol (Fluka, Saint-Quentin Fallavier, France) to obtain a culture of synchronous stationary-phase yeast. A loopful of this 48 h-culture was transferred to 25 mL of Yeast Nitrogen Medium (YNB; BioChemika Sigma Aldrich, Saint-Quentin Fallavier, France) supplemented with 30 mM of glucose (Sigma Aldrich) (YNB-Glc 30 mM) and incubated for 16 h at 37°C without shaking. Blastospores were then harvested and washed twice in phosphate-buffered saline (PBS; pH 7.2) and adjusted to the desired concentration.

### Carvacrol derivatives preparation

**Figure 1:**
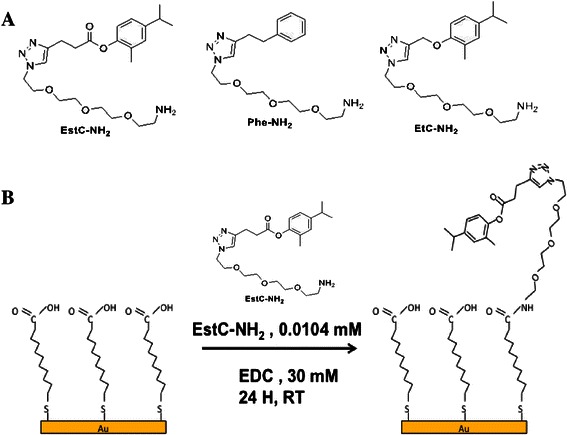
**Structures of the carvacrol derivatives grafted on gold surfaces (A). **Schematic representation of the two consecutive steps leading to the immobilization of EstC-NH_2_ on Au surface, via esterification of the acidic functions of MUA by EDC **(B)**.

EstC-NH_2_, Phe-NH_2_ and EtC.-NH_2_ (Figure 1A) were synthesized as described in the Additional file 1.

### Functionalization of gold samples

#### Grafting steps

The surfaces cited below as gold surfaces were purchased from Arrandee (Werther, Germany). They were constituted of glass substrates (11 × 11 × 1 mm) and coated successively with a 50 Å thick layer of chromium and a 200 nm thick layer of gold. The gold-coated substrates were annealed in a butane flame to ensure a good cristallinity of the topmost layers and rinsed first in a bath of absolute ethanol for 5 min and then twice in milliQ water for 5 min with shaking. The substrates were immersed in 10 mL of a 0.01 M solution of MUA in absolute ethanol for 3 h, to ensure an optimal homogeneity of the adlayer (Briand et al. 2006a; Briand et al. 2006b), and thoroughly rinsed in ethanol and water, and dried under a flow of dry nitrogen. Activation of acid groups into ester groups (EDC at 30 mM in water) and immobilization of EstC-NH_2_, Phe-NH_2_ or EtC.-NH_2_ (1.04 × 10^−2^ mM in water respectively corresponding to 4.5 mg/L, 3.5 mg/L and 4 mg/L) on gold surfaces was carried out in one step, without any catalyst, by depositing a 150 μL drop on the MUA-Au-modified substrates at room temperature for 24 h. After the immobilization step, the surfaces were vigorously rinsed in water under shaking for 5 min and finally dried under a flow of dry nitrogen (see Figure 1B for an illustration of the two grafting steps).

#### PM-RAIRS measurements

The gold samples were placed in the external beam of an FT-IR instrument (Nicolet Nexus 5700 FT-IR spectrometer), and the reflected light was focused on a nitrogen-cooled Mercury-Cadmium-Telluride wide band detector. The infrared spectra were recorded at 8 cm^−1^ resolution, with co-addition of 128 scans. A ZnSe grid polarizer and a ZnSe photoelastic modulator to modulate the incident beam between p and s polarizations (HINDS Instruments, PEM90, modulation frequency = 36 kHz) are placed prior to the sample. The detector output was sent to a two-channel electronic device that generates the sum and difference interferograms. Those are processed and undergo Fourier transformation to produce the ‘Polarization Modulation – Reflection Absorption Infrared Spectroscopy’ (PM-RAIRS) signal (ΔR/R^0^) = (Rp − Rs)/(Rp + Rs). Using a modulation of polarization enabled us to perform rapid analyses of the sample after treatment in various solutions without purging the atmosphere or requiring a reference spectrum.

#### XPS measurements

XPS analyses were performed using a PHOIBOS 100 X-ray photoelectron spectrometer from SPECS GmbH (Berlin, Germany) with a MgKa X-ray source (hn ¼ 1253.6 eV) operating at P = 1 × 10^−10^ Torr or less. Spectra were carried out with a 20 eV pass energy for the survey scan and 10 eV pass energy for the C1s, O1s, S2p and N1s regions. High-resolution XPS conditions have been fixed: “Fixed Analyser Transmission” analyses mode, a 7 × 20 mm entrance slit; leading to a resolution of 0.1 eV for the spectrometer, and an electron beam power of 150 W (12.5 kV and 12 mA). Such a low energy was used to keep the adsorbed layers as intact as possible. A takeoff angle of 90° from the surface was employed for each sample and binding energies were calibrated against the Au4f binding energy of the gold surface at 84.0 eV. Element peak intensities were corrected by Scofield factors (Scofield 1976). The spectra were fitted using the Casa XPS v.2.3.13 Software (Casa Software Ltd., UK) and applying a Gaussian/Lorentzian ratio, G/L equal to 70/30. In addition, the error in the peak fitting process was estimated at 5% or less with respect to the integration of the raw data.

### Adhesion of yeasts on gold samples

#### Adhesion phase of yeast cells

A Petri dish was partially filled with approximately 7 mL of a kappa carrageenan gel (15 g L^−1^; Sigma Aldrich) and cooled at room temperature. One gold sample (functionalized or control) was then carefully deposited per dish, the functionalized gold face upwards. A 100 μL drop containing 10^5^ of freshly cultured yeast cells resuspended in PBS was deposited on the gold surface. After 3 h at 37°C, each sample was washed three times in physiological sterile solution (0.9% NaCl).

#### Enumeration of adherent yeast cells

After the 3 h adhesion phase and the washing step, the gold surfaces were transferred into a sterile tube containing 2 mL of physiological sterile solution (0.9% NaCl), then sonicated 3 min at 60 W. After the removing of the gold sample, the yeast cells were pelleted by centrifugation at 13 000 *g* for 5 min. Then, 1.8 mL of supernatant was removed cautiously, and the cells were resuspended by vortexing. The suspension was then diluted 50 and 100 times; 50 μL of each dilution were plated on Sabouraud agar plates, in duplicate for each dilution, using a spiral plater WASP (AES, France). The plates were incubated at 37°C for 24 h before counting the colonies.

#### Microscopic analysis

Viability of adherent yeasts to the surfaces was evaluated with the LIVE/DEAD® Bacterial Viability Kit (BacLight®). Syto9 (at 2.34 mM) and propidium iodide (at 20 mM). The two stains, were diluted 10 times, in physiological sterile solution (0.09% NaCl) and were kept as stock solutions at −20°C, in the dark. The final BacLight solution used for the microscopic observations was freshly prepared by mixing 1.5 μL of both stain stock solutions with 97 μL of distilled water. After the 3 h adhesion phase and the washing step described above, a 10 μL drop of this BacLight mixture was deposited on the surface of the sample, a slide was put on the drop and the whole set was incubated for 15 min in the dark, at room temperature. Samples were then examined with a confocal FV-1000 station installed on an inverted microscope IX-81 (Olympus, Tokyo, Japan). Images were acquired with an Olympus UplanSapo × 60 water, 1.2 NA, objective lens (800 × 800 pixels images with 0.13 mm per pixel corresponding to Nyquist criteria for optimal sampling). Multiple fluorescence signals were acquired sequentially to avoid cross talk between image channels. Fluorophores were excited with the 488 nm line of an argon laser (for Syto9) and the 543 nm line of an HeNe laser (for propidium iodide). The emitted fluorescences were detected through spectral detection channels between 500–530 and 555–655 nm, for green and red fluorescence, respectively. Several photographs (from 5 to 10), of different areas with same surfaces, were taken on the same sample. Adherent yeast cells and adherent permeabilized (red) cells were manually counted and average numbers were calculated.

### Activity of already used surfaces

To investigate the persistence of the antifungal activity of already used Au-MUA-NH-EstC surfaces, microbial viability after adherence was assayed after 1 month's storage at 4°C by yeast numerations on plates and confocal microscopy analysis, using the same protocols described previously.

## Results

### EstC-NH_2_ molecule grafting and surface characterization

#### PM-RAIRS analysis

**Figure 2:**
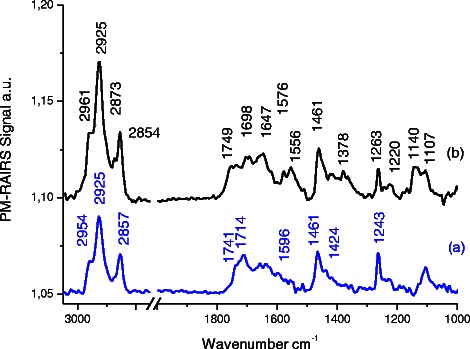
**PM-RAIRS spectra of the two consecutive steps leading to the immobilization of EstC-NH**_**2**_ **: (a) Au–MUA; (b) covalent binding of EstC-NH**_**2**_ **to MUA SAMs on gold surface.**

Spectrum (a) on Figure 2, which corresponds to the grafting of MUA on the gold plate (Au-MUA), presents the typical infrared signature of MUA (Bain et al. 1989; Tielens et al. 2008). All usual IR features are present on the spectrum: a broad peak with two main frequencies at 1741 and 1714 cm^−1^ assigned to the νC = O of the carboxylic acid group of the acidic thiolate; The presence of two frequencies for the same group suggests either two different adsorption sites and/or a poor order of the SAM. This is confirmed by the average order defined by the νCH_2_ at 2925 and 2854 cm^−1^ (highly ordered layer usually observed at 2919 and 2848 cm^−1^). In addition, two bands at 1596 and 1424 cm^−1^, assigned respectively to the anti-symmetric and symmetric νCOO−, suggest the presence of deprotonated carboxylate end groups (Bertilsson and Liedberg 1993; Briand et al. 2006a; Briand et al. 2006b). Thus, acidic functions are present under protonated and deprotonated chemical forms, both capable of reacting with EDC during the esterification process. In addition, the symmetric and antisymmetric stretching modes of the CH_2_ groups of the back bone of the thiol molecule are clearly visible at 2925 and 2854 cm^−1^, respectively. Eventually, PM-RAIRS spectra present bands related to the scissor mode of CH_2_ groups at 1461 cm^−1^ and to the stretching of C–OH of protonated COOH moieties at 1243 cm^−1^. Finally, there are some signs of residual contaminations in the MUA self-assembled monolayers, with the presence of a weak band at 2954 cm^−1^ (usually assigned to CH_3_ groups).

Spectrum (b), corresponding to the Au-MUA sample grafted with EstC-NH_2_, exhibits differences with respect to the MUA layer, suggesting some reactions after 24 h of immersion in a solution containing EDC + EstC-NH_2_. The binding of EstC-NH_2_ is indicated by the presence of new IR features in the 1500 and 1750 cm^−1^ region. Intense peaks at 1647 and 1556 cm^−1^ are assigned to the amide I and amide II bands of the peptidic backbone formed between the activated COOH group and the NH_2_ group of the EstC-NH_2_ molecule. The grafting of EstC-NH_2_ is also confirmed by the signature of the backbone of the molecules, mainly, the δOH at 1378 et 1263 cm^−1^, ωCH2 at 1461 cm^−1^, νC = O at 1698 and 1749 cm^−1^, νCH2 and νCH3 symmetric and antisymmetric at 2961, 2925, 2873 et 2854 cm^−1^, and probably the νC = C of the benzoic cycle à 1576 cm^−1^ also confirmed the successful grafting of the EstC-NH_2_ onto the MUA-SAMs on gold surface.

#### XPS analysis

**Figure 3:**
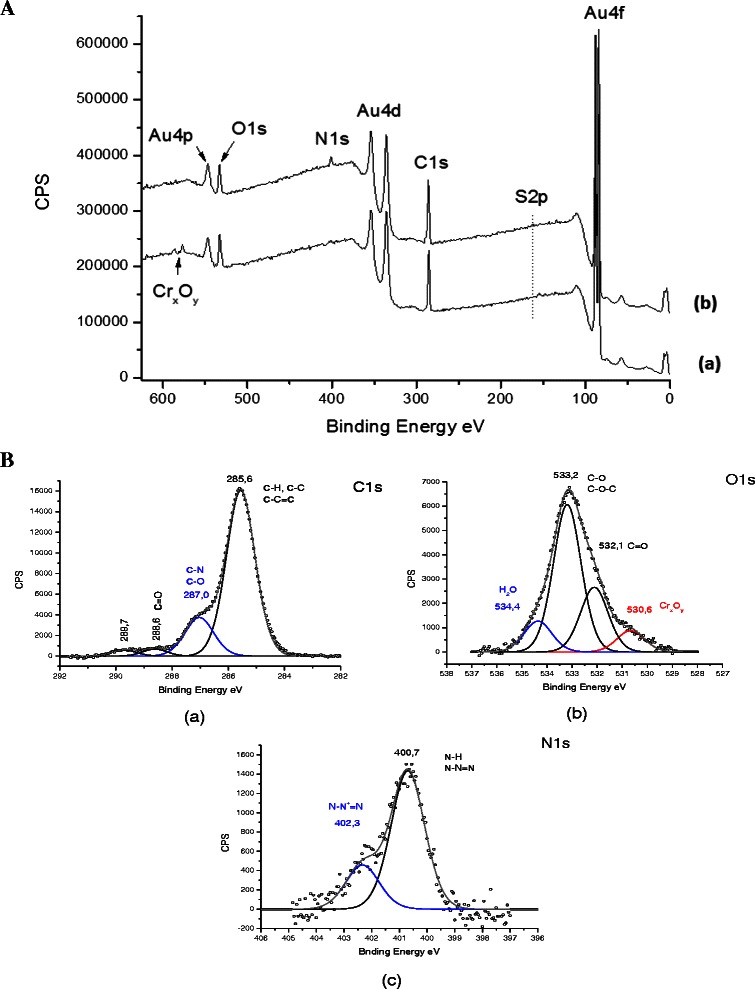
**XPS general spectra of gold surfaces.** **A:** XPS general spectra of gold surfaces functionalized (a) only with MUA (control) or (b) with the EstC-NH_2_ molecule. **B:** XPS high-resolution spectra of gold EstC-NH_2_ functionalized-surfaces for Carbon (a), for Oxygen (b) and for Nitrogen (c).

The general spectrum obtained by XPS spectroscopy as shown on Figure 3A for the gold surface functionalized with the EstC-NH_2_, reveals a signal at ~400 eV corresponding to nitrogen (indicated on the figure as N1s) that is not present on the Au-MUA control (spectrum a), clearly suggesting the presence of the EstC-NH_2_ molecules at the surface.

As shown on Table 1, the experimental percentages corresponding to the C, O, N and S elements are in good agreement with the estimated theoretical percentages. Sulphur is slightly under-estimated on the experimental gold surface, probably because of its position on the surface (see Figure 1). Nitrogen is also under-estimated for the benefit of carbon, maybe due to the molecular adsorption geometry undertaken by the EstC-NH_2_ molecules when chemically grafted on the surface.

**Table 1:** **Comparison between theoretical and experimental (XPS) percentages for C, O, N and S atoms, for the surface after both functionalization steps (Au_MUA-EstC-NH**_**2**_ **)**

**Atomic percentages**	**C**	**O**	**N**	S
Theoretical	75.6%	13.3%	8.9%	2.2%
Experimental	81.0%	13.4%	4.3%	1.3%

When looking at the high resolution XPS spectra of the mains elements, Core level C1s, O1s and N1s), the successful grafting of the molecules is confirmed. On the C1s XPS spectrum (Figure 3B (a)), all the expected components are found, in particular, the main peak shoulder at 287.0 eV, characteristic of C-O-C bindings that is quite represented in the EstC-NH_2_ molecule. This signature also appears on the oxygen spectrum (Figure 3B (b)), with a key component at 533.2 eV. Finally, the nitrogen spectrum (Figure 3B (c)) is defined by two different components, one at 400.7 eV characteristics of both amide bindings (N-H) and the different nitrogen atoms on the unsaturated triazole cycle. The second contribution at 402.3 eV is not easily assignable and could be associated to a quaternary ammonium displaying a positive charge of the molecule.

The EstC-NH_2_ grafting on the thiol MUA spacer through the formation of an amide binding was confirmed by the PM-RAIRS and XPS complementary experiments.

### Antimicrobial activities of EstC-NH_2_ functionalized gold samples

#### Culturability of Candida albicans after adherence on EstC functionalized gold samples

**Figure 4:**
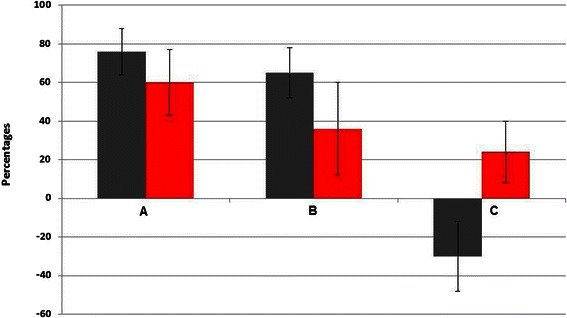
**Percentages of inhibition of the culturability of** ***Candida albicans*** **after a 3 h of incubation on the different functionalized gold samples, A = EstC-NH**_**2**_ **, B = EtC.-NH**_**2**_ **and C = Phe-NH**_**2**_ **compared to the control MUA (grey) and percentages of the number of red stained cells compared to the control MUA (LIVE/DEAD® Bacterial Viability Kit (BacLight®) (red).**

The culturability of adherent *C. albicans* ATCC 3153 on EstC-NH_2_ functionalized gold surfaces was inhibited by 76% ± 11% compared to the control Au-MUA (Figure 4A, n = 4). The experiment duration of 3 h is not long enough to permit biofilm formation.

#### Microscopic analysis

**Figure 5:**
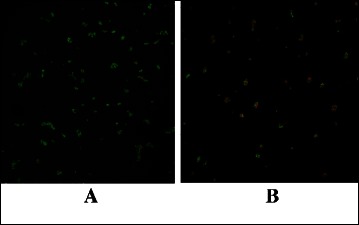
**Viability of adherent** ***C. albicans*** **ATCC 3153, revealed by BacLight™ stain, attached to gold surfaces grafted with MUA (A) or with EstC-NH**_**2**_ **(B) observed by confocal microscopy (magnification × 20).** 10^6^ Candida cells were deposited on the samples and allowed to adhere for 3 h.

Confocal microscopy analyses were carried out using BacLight™ staining. Briefly, the cellular membrane of the yeasts which appeared red is damaged, whereas green cells have maintained their integrity and are considered as alive. Indeed, the red stain (PI) can only penetrate permeabilized cells, whereas the green one (Syto9) can cross over intact cellular membranes. Images obtained by confocal microscopy analyses are presented in Figure 5. Entire surfaces of three samples of each type of substrate were analysed. On the control surface, grafted with MUA only, *C. albicans* ATCC 3153 cells appeared as small green clusters corresponding to living yeasts (Figure 5A). As the yeast density differs according to the surface area that is observed under the microscope, several pictures were taken on the same surface and a mean density of each type of stained cells, red and green, was calculated for each surface after having manually counted both Syto9- and PI-stained cells. On the surface functionalized with EstC-NH_2_, the number of total adherent cells was similar to the control Au-MUA but the number of red permeabilized and probably dead cells, increased by 60% comparing to the same control (Figure 4A and Figure 5B) (n = 2).

### EtC.-NH_2_ molecule grafting and surface characterization

#### PM-RAIRS analysis

**Figure 6:**
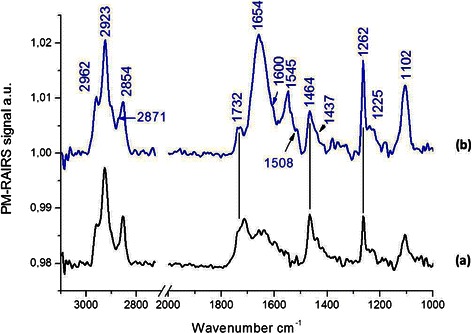
**PM-RAIRS spectra of the two consecutive steps leading to the immobilization EtC.-NH**_**2**_ **.** Spectrum (a) represents the control Au–MUA and spectrum (b) is associated to the covalent binding of the CAR derivative.

Considering the spectrum b on Figure 6, EtC.-NH_2_ was successfully grafted onto the MUA-SAMs on gold surface. Indeed the amid I and II bands (νC = O and δNH, respectively) are found at 1654 and 1545 cm^−1^, showing the covalent bound formed between the activated COOH group and the NH_2_ group of the EtC.-NH_2_ molecule. On the same spectrum, one can observe the vibrations assigned to the Ether, with the νC = C and the νN = C-N respectively at 1600 cm^−1^ and 1508 cm^−1^ (also with a “shoulder” at 1545 cm^−1^) and both νC-O-C symmetric and anti-symmetric stretching mode are found at 1102 cm^−1^ and at 1262 et 1225 cm^−1^.

No further XPS experiments were performed at this stage as the comparison of PM-RAIRS results of EtC.-NH_2_ with those of EstC-NH_2_ was sufficient.

### Antifungal activity and mode of action of EtC.- functionalized gold surfaces

The number of adhered culturable C. albicans ATCC 3153 was inhibited by 65% ± 13% on EtC.-functionalized gold surfaces compared to the control Au-MUA (Figure 4B; n = 4).

**Figure 7:**
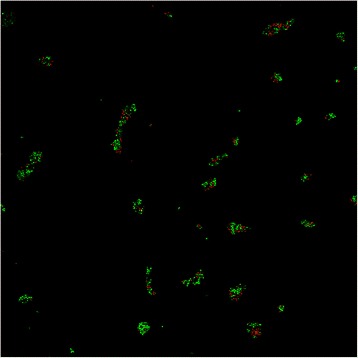
**Viability of** ***C. albicans*** **ATCC 3153, revealed by BacLight™ stain, attached to gold surfaces grafted with EtC.-NH**_**2**_ **, observed by confocal microscopy (magnification × 20).** 10^6^ Candida cells were deposited on the samples and allowed to adhere for 3 h.

On the surface functionalized with EtC.-NH_2_, the number of total adherent cells was similar to the control Au-MUA but the number of red permeabilized and probably dead cells increased by 36% comparing to the MUA control as shown on Figure 7 (n = 2) (See also Figure 4B).

### Complementary functionalization of gold surfaces and its antifungal activity associated to the Phe-NH_2_ molecule

#### PM-RAIRS

**Figure 8:**
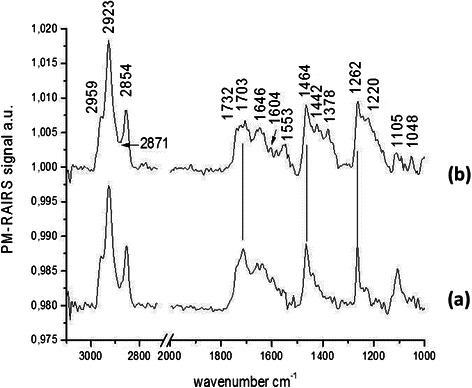
**PM-RAIRS spectra of the two consecutive steps leading to the immobilization Phe-NH**_**2**_ **.** Spectrum (a) represents the control Au–MUA and spectrum (b) is associated to the covalent binding of the CAR derivative.

On spectrum b, on Figure 8, corresponding to the Phe-NH_2_ molecule, the amid I and II bands (νC = O and δNH, respectively) are found at 1646 and 1553 cm^−1^ showing the covalent bound formed between the activated COOH group and the NH_2_ group of the designed molecule. Moreover, the vibrations associated to the triazole cycle are observed, namely the νC = C at 1604 cm^−1^ and the weak bands νN = C-N at 1518 cm^−1^ (with a “shoulder” at 1533 cm^−1^). Finally the characteristic νC-O-C symmetric and antisymmetric vibrations of the backbone of the molecules are found on one hand at 1105 and 1048 cm^−1^ and at 1262 and 1220 cm^−1^ on the other hand. PM-RAIRS measurements confirmed the grafting of Phe-NH_2_ molecule.

### Antifungal activity and mode of action of Phe - functionalized gold surfaces

The number of adhered culturable C. albicans ATCC 3153 was enhanced by a mean of 30% on Phe-functionalized gold surfaces (Figure 4C; n = 4) and not inhibited as in the cases of the two CAR derivatives.

**Figure 9:**
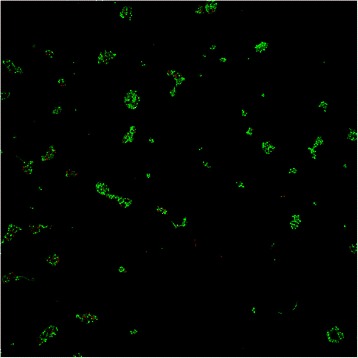
**Viability of** ***C. albicans*** **ATCC 3153, revealed by BacLight™ stain, attached to gold surfaces grafted with Phe-NH**_**2**_ **, observed by confocal microscopy (magnification × 20).** 10^6^ Candida cells were deposited on the samples and allowed to adhere for 3 h.

On the surface functionalized with the Phe-NH_2_ molecule, the number of adherent cells was similar to the control Au-MUA but the number of red permeabilized and probably dead cells increased by only 24% comparing to the MUA control as shown on Figure 9 (n = 2) (See also Figure 4C).

### Activity of already used surfaces

To investigate the persistence of the antifungal activity of already used Au-MUA-NH-EstC surfaces, microbial viability after adherence was assayed after 1 month's storage at 4°C, using the same protocol as above. Samples were washed in pure water, dried under nitrogen, and stored at 4°C before the second assay. The culturability of adherent *C. albicans* ATCC 3153 on already-used EstC-NH_2_ functionalized gold surfaces was still inhibited by 80% ± 13% compared to the control Au-MUA (10^6^*Candida* cells were deposited on the samples and allowed to adhere for 3 h; n = 2). On confocal microscopy pictures of already-used EstC-NH_2_ surfaces, the number of total adherent cells was similar to the control Au-MUA but the number of red permeabilized and probably dead cells increased by 68% comparing to the same control (n = 2).

In order to estimate the amount of released CAR from the EstC-NH_2_ functionalized surfaces, previously used surfaces were further analysed by PM-RAIRS, XPS and AFM. These assays revealed that there is nearly no loss of CAR on the surface after having been in contact with yeast cells (data not shown).

## Discussion

The idea behind this study was to anchor the volatile CAR on a surface in order to stabilize it while keeping its interesting antimicrobial/antifungal activity. Thus, two new molecules deriving from this terpene were specifically synthesized to be grafted on gold surfaces. They were named “Carvacrol Ester” or EstC-NH_2_ and “Carvacrol Ether” or EtC.-NH_2_. These two molecules both include a spacer which function is to take the active part of the molecule containing CAR away from the gold surface to increase its accessibility. A triazole cycle is present in the spacer structure. This triazole cycle results from the chemistry used to synthesize the EstC-NH_2_ molecule (*i.e.* The Click Chemistry between an alkyne function and an azide function). The terminal part of the two constructs includes CAR which is linked to the rest of the molecule through an ester bond or an ether one. The first postulate of this work relied on the fact that the ester bond could be broken by esterase enzymes from microorganisms (*i.e.* yeasts or bacteria) arriving on the functionalized surface, thus allowing the liberation of CAR and its antifungal/antimicrobial activity. It is supposed to allow a customized release of CAR which should avoid a quick exhaustion of CAR on the surface or a loss of its activity often induced by a permanent binding. After CAR release, it could exert its antimicrobial activity on microorganisms, adherent or not, that are in its surrounding environment.

The other objective of this work was to design surfaces to which CAR is covalently attached and to study their antifungal activity. Once the functionalization of gold surface with EstC-NH_2_ performed and checked by PM-RAIRS measurements, the inhibition of culturability of adherent *C. albicans* ATCC 3153 on these surfaces reached 76% ± 11% compared to the control Au-MUA. This decrease in the number of culturable cells attached to the functionalized surfaces can be explained by two processes. Either the grafting of EstC-NH_2_ makes the surfaces antiadhesive thus preventing the yeasts from attaching onto the substrates, or it exerts an antifungal activity toward cells after their adhesion via a fungicidal or fungistatic mode of action. To answer this question, confocal microscopy analyses, using BacLight™ staining showed that on the surface functionalized with EstC-NH_2_, the number of adherent cells was similar to the control Au-MUA but the number of red permeabilized cells, increased by more than 60%. The mode of action of these newly-developed EstC-NH_2_ functionalized surfaces appears to be rather fungicidal but not antiadhesive.

In the same way, the loss of culturability on EtC.-NH_2_ functionalized gold surfaces was 65% ± 13% and 36% more cells appeared red comparing to the MUA control. Thus, the mode of action of these EtC.-NH_2_ functionalized surfaces appears to be fungicidal but less than the EstC-NH_2_ functionalized one.

Consequently, the antifungal activity of both EstC-NH_2_ and EtC.-NH_2_ functionalized surfaces certainly results from the CAR molecule. This statement was reinforced by the experiments on Phe-NH_2_ functionalized surfaces which did not impair the yeast culturability. Moreover, the activity seems to be mainly due to still fixed CAR that is not released by the action of esterases. As shown by PM-RAIRS, XPS and AFM experiments on previously used EstC-NH_2_ functionalized surfaces*,* there is nearly no loss of CAR on the surface having been in contact with yeast cells (data not shown). As a consequence, it could be stated that CAR does not required to be released from the surface to exert its antifungal activity.

At this stage of the study, another question emerged concerning the process that governs the interesting fungicidal activity recorded for the two types of surfaces functionalized with CAR derivatives. Is this activity directly linked to the antifungal activity of CAR or is it due to the presence of the triazole cycle in the EstC and EtC. molecule backbones? Indeed, triazole moieties are essential components of the molecules that belong to the triazole class, a major group of systemic antifungals (Odds et al. 2003). However the non-activity of Phe-NH_2_ functionalized surfaces demonstrated that the triazole cycle is not antifungal in this specific case.

Taking in consideration the methodology applied to prepare the surfaces and the possibility of having performed esterification and etherification reactions with a limited yield, it is possible that the grafting reaction does not cover the full treated surface. Therefore it could be considered that the obtained surfaces were not fully homogeneous, leading to domains free of CAR within the samples. Indeed, the confocal microscopy images displayed in Figures 5, 7 and 9 might indicate the formation of clusters of living (or dying) cells but without interconnection between colonies that could be in correlation with the heterogeneous distribution of CAR among the surface.

The persistence of the antifungal activity of already used Au-MUA-NH-EstC surfaces was verified after 1 month's storage at 4°C. Indeed, the culturability of adherent *C. albicans* was still inhibited by 80% ± 13% and confocal microscopy photographs, revealed that the number of red cells on already-used EstC-NH_2_ surfaces increased by 68% comparing to the control. Thus, the antifungal and fungicidal activity of the newly-functionalized gold surfaces with the EstC-NH_2_ molecule is conserved once used and sonicated even after being stored one month at 4°C which is a promising and desired property. Similar results were obtained previously for surfaces grafted with antimicrobial peptides Magainin and Gramicidin (Humblot 2009, Yala 2011) indicating that, as expected, the covalent grafting of antimicrobial molecules extends the shelf-life of antimicrobial materials.

To conclude, this proof of concept work demonstrated that gold surfaces grafted with carvacrol (CAR) integrated in specifically designed constructs displayed a promising antifungal activity. Confocal microscopy analyses highlighted that the newly-developed surfaces mode of action was fungicidal. It was shown, using a phenyl analogue replacing CAR as well as by replacing the ester bond by an ether linkage in the derivative structure, that the antifungal activity was directly linked to CAR. This result as well as the subsequent surface analysis of already-used functionalized gold samples indicated that the antifungal activity was finally not related to the cleavage of the ester bond and to the resulting release of CAR. Interestingly, the activity of the already-used grafted surfaces even persisted after the sonicating and washing steps. This study revealed that the immobilization of antimicrobial aromatic terpenic components, such as carvacrol, involved in an innovative chemical structure designed to overcome their high volatility and thus the resulting limits to their use, can considerably inhibit yeast adhesion and subsequently delay biofilm formation. The developed surfaces were first tested on *C. albicans.* As terpenes are known to exert both an antimicrobial and antifungal activity, one next step will be to further investigate the activity of these new functionalized samples on other *Candida* and yeast species but also on bacteria using experimental conditions mimicking real biological conditions. Prospects before making these new surfaces usable in clinical practice are many.

## Additional file

Additional file 1:
**Carvacrol derivatives preparation.**
