# Erratum to: Surface functionalization by covalent immobilization of an innovative carvacrol derivative to avoid fungal biofilm formation

**DOI:** 10.1186/s13568-015-0141-4

**Published:** 2015-08-21

**Authors:** Aïcha Gharbi, Thibaut Legigan, Vincent Humblot, Sébastien Papot, Christine Imbert, Jean-Marc Berjeaud

**Affiliations:** Ecologie and Biologie des Interactions-UMR CNRS 7267, Microbiologie de l’eau, Université de Poitiers, 1 Rue Georges Bonnet, TSA 51106, 86073 Poitiers Cedex 9, France; UMR-CNRS 7285, Groupe Systèmes Moléculaires Programmés, Université de Poitiers, Poitiers, France; UPMC Université Paris 06, UMR CNRS 7197, Laboratoire de Réactivité de Surface, Sorbonne Universités, Paris, France

## Erratum to: AMB Express (2015) 5:9 DOI: 10.1186/s13568-014-0091-2

The original version of this article (Gharbi et al. 2015) unfortunately contained a mistake. Dr. Christine Imbert and associated affiliation was missing from the author list in the HTML and PDF versions of this article. The corrected author list is given below.

Aïcha Gharbi^1^, Thibaut Legigan^2^, Vincent Humblot^3^, Sébastien Papot^2^, Christine Imbert^1^ and Jean-Marc Berjeaud^1^

In addition, the following information was missing from the acknowledgements section in both the PDF and HTML versions of this manuscript:

This study was partially supported by a research Grant from Pfizer.
