# Supplementary material for: Surface functionalization by covalent immobilization of an innovative carvacrol derivative to avoid fungal biofilm formation
Source: AMB Express. 2015 Feb 5;5:9. doi: 10.1186/s13568-014-0091-2 (PMC4384722; doi:10.1186/s13568-014-0091-2)
Supplement: Additional file 1: — Carvacrol derivatives preparation. [file 13568_2014_91_MOESM1_ESM.docx]

***Carvacrol derivatives preparation***

Scheme S1 **Reagents and conditions : (**i) N-hydroxysuccinimide, N-(3-dimethylaminopropyl)-N’-ethylcarbodiimide hydrochloride, CHCl_3_/DMF 9/1, 24h, rt, 90%; (ii) carvacrol, 4-(dimethylamino)pyridine, 1,2-dichloroethane, 48h, 60°C, 76%; (iii) tetrakis(acetonitrile)copper(I) hexafluorophosphate, **3**, CH_2_Cl_2_, 17h, rt, 79%; **(**iv) tetrakis(acetonitrile)copper(I) hexafluorophosphate, **3**, CH_2_Cl_2_, 17h, rt, 34%; (v) propargyl bromide, K_2_CO_3_, DMF, 6h, 80°C, 92% (vi) tetrakis(acetonitrile)copper(I) hexafluorophosphate, **3**, CH_2_Cl_2_, 24h, rt, 48%.

Pure CAR solution (5-Isopropyl-2-methylphenol) (Purity >= 98%) was purchased from Sigma Aldrich and kept at 4 °C. CAR working-solutions in methanol were freshly prepared before use from a stock solution at 10.7 x 10^3^ mM. Stock solutions of CAR derivatives: EstC-NH_2_, Phe-NH_2_ and EtC-NH_2_ were prepared in DMSO at respectively 569 mM, 731 mM and 640 mM (corresponding for the three molecules to a concentration of 256 g/L) and stored at -20°C. 11-mercaptoundecanoïc acid (MUA) and 1-(3-dimethylaminopropyl)-N′-ethylcarbodiimide hydrochloride (EDC) were purchased from Sigma Aldrich. All solvents were reagent grade. Reagents were used without any further purification. Experiments were carried out at room temperature if not specified otherwise. All reactions were performed under a nitrogen atmosphere. Unless otherwise stated, solvents used were of HPLC quality. Chemicals were of analytical grade from commercial sources and were used without further purification.

The reaction progress was monitored on precoated silica gel TLC plates MACHEREY-NAGEL ALUGRAM® SIL G/UV254 (0.2 mm silica gel 60). Spots were visualized under 254 nm UV light and/or by dipping the TLC plate into a solution of 3 g of phosphomolibdic acid in 100 mL of ethanol followed by heating with a hot gun. Flash column chromatography was performed using MACHEREY-NAGEL silica gel 60 (15-40 µm) as the stationary phase. ^1^H and ^13^C NMR spectra were recorded on a Bruker 400 Avance III instrument, equipped with an ultra-shielded magnet and a BBFO 5 mm broadband probe (NMR spectra of the different compounds can be found in the appendices). High resolution ESI mass spectrometry was carried out by the CRMPO (“Centre Régional de Mesures Physiques de l’Ouest”), at the University of Rennes 1.

EstC-NH_2_, Phe-NH_2_, and EtC-NH_2_ and all intermediate compounds were prepared according to the following strategies (Scheme S1). Azido-PEG **3** was synthesized as described in the literature ([Schwabacher et al. 1998](#_ENREF_1)).Chemical shifts (δ) are reported in parts per million (ppm) from high to low field and referenced to residual solvent. Coupling constants (*J*) are reported in hertz (Hz). Standard abbreviations indicating mutliplicity are used as follows: b = broad, s = singlet, d = doublet, t = triplet, dd = doublet of doublets dt = doublet of triplets, m = mutliplet.

*Compound 1.*

To a solution of 4-pentynoic acid (100 mg; 1.02 mmol) and *N*-hydroxysuccinimide (117 mg; 1.02 mmol) in CH_3_Cl/DMF 9/1 (1 mL) was added EDC.HCl (181 mg; 1.02 mmol). The mixture was stirred 24h at room temperature. The crude reaction mixture was diluted with CH_2_Cl_2_ (50 mL) and washed with saturated aqueous NaHCO_3_ (3🞪50 mL). The organic layer was dried over MgSO_4_, filtrated and concentrated *in-vacuo* to give **1** (180 mg; 0.92 mmol; 90% yield) as a white solid which was used without further purification. ^1^H NMR (400 MHz, CDCl_3_) δ: 2.06 (t, 1H, *J* = 2.6 Hz), 2.63 (dt, 2H, *J* = 2.6 Hz, *J* = 7.0 Hz), 2.85 - 2.91 (m, 6H).

*Compound 2.*

Carvacrol (150 mg; 1 mmol) and NHS ester **1** (293 mg; 1.5 mmol) were dissolved in dry 1,2-dichloroethane (10 mL). DMAP (240 mg; 2 mmol) was added and the mixture was stirred for 48h at 60°C. The crude reaction mixture was diluted with CH_2_Cl_2_ (40 mL) and washed with NaHCO_3_ (3🞪50 mL) and aqueous 1M NaOH (3🞪50 mL). The organic layer was dried over MgSO_4_, filtered and concentrated *in-vacuo*. Purification *via* column chromatography over silica gel (hexanes/AcOEt 98/2) afforded the pure product as an oil (174 mg; 0.76 mmol; 76% yield). ^1^H NMR (400 MHz, CDCl_3_) δ: 1.23 (d, 6H, *J* = 6.9 Hz), 2.04 (t, 1H, *J* = 2.6 Hz), 2.15 (s, 3H), 2.65 (dt, 2H, *J* = 2.6 Hz, *J* = 7.0 Hz), 2.82 – 2.91 (m, 3H), 6.87 (d, 1H, *J* = 1.7 Hz), 7.02 (dd, 1H, *J* = 1.7 Hz, *J* = 7.8 Hz), 7.15 (d, 1H, *J* = 7.8 Hz). ^13^C NMR (100 MHz, CDCl_3_) δ: 14.7, 16.0, 24.0 (2C), 33.6, 33.7, 69.6, 82.3, 119.8, 124.3, 127.2, 131.0, 148.2, 149.2, 170.2. HRMS ESI [M+Na]^+^ *m/z* 253.1203 (theoretical for C_15_H_18_O_2_Na: 253.1204).

*EstC-NH2.*

Tetrakis(acetonitrile)copper(I) hexafluorophosphate (225 mg; 0.651 mmol) was added to a solution of carvacrol ester **2** (100 mg; 0.43 mmol) and azido-PEG **3** (85 mg; 0.391 mmol) in dry CH_2_Cl_2_ (5 mL). The mixture was stirred for 17h at room temperature and a saturated solution of disodium EDTA in 0.2 phosphate buffer (10 mL) was added. The mixture was stirred at room temperature for 3h and extracted with CH_2_Cl_2_ (6🞪30 mL). The organic layer was dried over MgSO_4_, filtered and concentrated *in-vacuo*. The crude product was purified by column chromatography over silica gel (CH_2_Cl_2_/MeOH 100/0 – 95/5) to afford **EstC-NH_2_** (141 mg; 0.31 mmol; 79% yield) as an oil. ^1^H NMR (400 MHz, CDCl_3_) δ: 1.19 (d, 6H, *J* = 6.9 Hz), 2.02 (s, 3H), 2.80 – 2.87 (m, 1H), 2.99 – 3.00 (m, 2H), 3.11 – 3.13 (m, 2H), 3.23 (m, 2H), 3.51 – 3.60 (m, 8H), 3.71 – 3.73 (m, 2H), 3.78 – 3.80 (m, 2H), 4.49 – 4.52 (m, 2H), 6.84 (s, 1H), 6.98 (dd, 1H, *J* = 1.6 Hz, *J* = 7.8 Hz), 7.09 – 7.11 (m, 3H), 7.61 (bs, 1H). ^13^C NMR (100 MHz, CDCl_3_) δ: 15.8, 20.8, 24.0 (2C), 33.2, 33.6, 40.4, 50.2, 66.5, 69.6 – 70.1 (5C), 117.0, 119.9, 124.3, 127,2, 131.0, 148.3, 149.2 (C_triazole_, C_ester_ were not observed). HRMS ESI [M+H]^+^ *m/z* 449.2769 (expected for C_23_H_37_N_4_O_5_: 449.2764)

*Phe-NH2.*

Tetrakis(acetonitrile)copper(I) hexafluorophosphate (291 mg; 0.840 mmol) was added to a solution of 4-phenyl-1-butyne (70 µL; 0.50 mmol) and azido-PEG **3** (110 mg; 0.50 mmol) in dry CH_2_Cl_2_ (6.5 mL). The mixture was stirred for 17h at room temperature and a saturated stirred at room temperature for 3h and extracted with CH_2_Cl_2_ (6🞪30 mL). The organic layer was dried over MgSO_4_, filtered and concentrated *in-vacuo*. The crude product was purified by column chromatography over silica gel (CH_2_Cl_2_/MeOH 100/0 – 90/10) to afford **Phe-NH_2_** (59 mg; 0.17 mmol; 34% yield) as an oil. ^1^H NMR (400 MHz, CDCl_3_) δ: 1.99 – 2.05 (m, 2H), 2.74 – 2.86 (m, 2H), 2.95 – 3.03 (m, 4H), 3.49 (t, 2H, *J* = 5.2 Hz), 3.57 – 3.51 (m, 8H), 3.83 (t, 2H, *J* = 5.2 Hz), 4.48 (t, 2H, *J* = 5.0 Hz), 7.16 – 7.29 (m, 5H), 7.37 (s, 1H). ^13^C NMR (100 MHz, CDCl_3_) δ: 27.4, 35.5, 41.5, 50.0, 65.5, 70.1, 70.3, 70.4 (2C), 73.1, 122.0, 126.0, 128.3, 128.4, 141.2, 147.0. HRMS ESI [M+H]^+^ *m/z* 349.2241 (theoretical for C_18_H_29_N_4_O_3_: 349.2240).

*Compound 4.*

To a solution of carvacrol (300 mg; 2 mmol) in dry DMF (6 mL) were added propargyl bromide 80% wt in toluene (323 µL; 3 mmol) and K_2_CO_3_ (414 mg ; 3 mmol). The reaction mixture was stirred for 6h at 80°C and diluted with water (30 mL). The mixture was extracted with CH_2_Cl_2_ (3🞪30 mL) and the organic layer was dried over MgSO_4_, filtered and concentrated *in-vacuo*. Purification *via* column chromatography over silica gel (hexanes/AcOEt 99.5/0.5) afforded the pure carvacrol ether **4** as an oil (347 mg; 1.84 mmol; 92% yield). ^1^H NMR (400 MHz, CDCl_3_) δ: 1.26 – 1.28 (m, 6H), 2.23 (s, 3H), 2.52 (t, 1H, *J* = 2.4 Hz), 2.87 – 2.94 (m, 1H), 4.73 (d, 2H, *J* = 2.4 Hz), 6.80 (d, 1H, *J* = 7.6 Hz), 6.84 (s, 1H), 7.09 (d, 1H, *J* = 7.6 Hz). ^13^C NMR (100 MHz, CDCl_3_) δ: 16.0, 24.2, 34.2, 56.1, 75.2, 79.2, 110.4, 119.2, 124.7, 130.8, 147.9, 155.8. HRMS ESI [M+Na]^+^ *m/z* 211.1097 (expected for C_13_H_16_ONa: 211.1099).

*EtC-NH2.*

Tetrakis(acetonitrile)copper(I) hexafluorophosphate (260 mg; 0.75 mmol) was added to a solution of carvacrol ether **4** (94 mg; 0.50 mmol) and azido-PEG **3** (109 mg; 0.50 mmol) in dry CH_2_Cl_2_ (6.5 mL). The mixture was stirred for 24h at room temperature and a saturated solution of disodium EDTA in 0.2 phosphate buffer (10 mL) was added. The mixture was stirred at room temperature for 1h and extracted with CH_2_Cl_2_ (6🞪30 mL). The organic layer was dried over MgSO_4_, filtered and removed under reduced pressure. The crude product was purified by column chromatography over silica gel (CH_2_Cl_2_/MeOH 96/4 – 92/8) to afford pure **EtC-NH_2_** (99 mg; 0.24 mmol; 48% yield) as an oil. ^1^H NMR (400 MHz, CDCl_3_) δ: 1.21 (2d, 6H, *J* = 6.9 Hz, two conformers), 2.17 (2s, 3H, two conformers), 2.81 – 2.89 (m, 3H), 3.24 (bs, 2H), 3.50 – 3.52 (m, 2H), 3.52 – 3.58 (m, 8H), 3.87 – 3.90 (m, 2H), 4.55 – 4.58 (m, 2H), 5.20 (2s, 2H, two conformers), 6.73 – 6.75 (m, 1H), 6.83 (s, 1H), 7.04 (dd, 1H, *J* = 2.6 Hz, *J* = 7.6 Hz), 7.80 (s, 1H). ^13^C NMR (100 MHz, CDCl_3_) δ: 16.0, 24.2 (2C), 34.1, 41.3, 50.4, 62.5, 69.6, 70.2, 70.5, 70.6 (2C), 71.9, 110.2, 118.7, 123.8, 124.3, 130.6, 144.7, 148.1, 156.5. HRMS ESI [M+H]^+^ *m/z*  407.2656 (expected for C_21_H_35_N_4_O_4_: 407.2658).

**NMR spectra for compound 1**

| **** |
| --- |

**NMR spectra for compound 2**

|  |
| --- |

| **** |
| --- |

**NMR spectra for EstC-NH_2_**

|  |
| --- |

| \|  \| \| --- \| |
| --- | --- |

**NMR spectra for Phe-NH_2_**

|  |
| --- |

|  |
| --- |

**NMR spectra for compound 4**

|  |
| --- |

|  |
| --- |

**NMR spectra for compound EtC-NH_2_**

|  |
| --- |

|  |
| --- |

Schwabacher AW, Lane JW, Schiesher MW, Leigh KM, Johnson CW (1998) Desymmetrization reactions: efficient preparation of unsymmetrically substituted linker molecules. J Org Chem 63:1727-1729.
